# Psychological violence against pregnant women in a prenatal care cohort: rates and associated factors in São Luís, Brazil

**DOI:** 10.1186/1471-2393-14-66

**Published:** 2014-02-12

**Authors:** Marizélia Rodrigues Costa Ribeiro, Antônio Augusto Moura da Silva, Maria Teresa Seabra Soares de Britto e Alves, Rosângela Fernandes Lucena Batista, Lourdes Maria Leitão Nunes de Rocha, Lilia Blima Schraiber, Nilzângela Lima Medeiros, Danielle Cristina Silva Costa, Heloisa Bettiol, Marco Antônio Barbieri

**Affiliations:** 1 Department of Medicine III, Federal University of Maranhão, Sao Luis, Brazil; 2 Department of Public Health, Federal University of Maranhão, Sao Luis, Brazil; 3 Department of Social Service, Federal University of Maranhão, Sao Luis, Brazil; 4 Faculty of Medicine, University of São Paulo, São Paulo, Brazil; 5 Postgraduation Program in Collective Health, Federal University of Maranhão, Sao Luis, Brazil; 6 Medical student, Federal University of Maranhão, Sao Luis, Brazil; 7 Faculty of Medicine of Ribeirão Preto, University of São Paulo, São Paulo, Brazil

**Keywords:** Prenatal care, Gender and health, Pregnant women, Violence against women, Domestic violence

## Abstract

**Background:**

Violence against pregnant women has been associated with gestational and perinatal disorders. Psychological violence is the type least investigated and its associated factors have been little studied. The present study was conducted in order to estimate prevalence rates and analyze the factors associated with exclusive and recurrent psychological violence in the municipality of São Luís, Brazil.

**Methods:**

Data regarding 982 pregnant women, aged from 14 to 45 years, interviewed in 2010 and 2011 in a prenatal cohort were used. A self-applied questionnaire was used to screen for violence. Pregnant women submitted to physical and sexual violence were excluded from the analysis of factors associated with exclusive psychological violence. Prevalence ratios and 95% confidence intervals were estimated by a Poisson regression model with a hierarchical approach at three levels. At level 1 of the theoretical-conceptual model, we analyzed demographic and socioeconomic characteristics and variables that express gender inequalities; at level 2, we analyzed social support received by the women, and at level 3, the life experiences of the pregnant women.

**Results:**

Prevalence rate of exclusive psychological violence was 41.6% and of recurrent violence was 32.6%. Exclusive psychological violence was associated with pregnant women’s age of 14 to 18 years (PR: 1.32 95% CI: 1.04 – 1.70), pregnant women’s schooling superior to that of her intimate partner (PR: 1.54 95% CI: 1.09 – 2.16), inadequate social affective support/positive social interaction (PR: 1.34 95% CI: 1.11 – 1.62), use of illicit drugs by the pregnant women (PR: 1.80 95% CI: 1.16 – 2.81) and having had six or more intimate partners in life (PR: 1.52 95% CI: 1.18 – 1.96). Recurrent exclusive psychological violence was associated with inadequate social affective support/positive social interaction (PR: 1.47 95% CI: 1.15 – 1.87), use of illicit drugs by the pregnant women (PR: 2,28 95% CI: 1,40 - 3,71) and having had six or more intimate partners in life (PR: 1.47 95% CI: 1.06 – 2.03).

**Conclusions:**

Psychological violence was a common phenomenon in this population of pregnant women that was associated with gender inequalities, inadequate social support and illicit drug use and should be routinely investigated during prenatal visits at health care services.

## Background

The expression *violence against women* has been defined as any gender-based action or conduct that causes death or physical, sexual or psychological damage to women both in public and private spheres [1].

Any act of violence against women must be considered as a violation of human rights and as a public health problem [2]. In addition to physical injuries and sexually transmitted diseases, which can be more easily imputed to aggression, other disorders such as depression, suicide, drug abuse, delayed beginning of prenatal care and preterm birth have been associated with maltreatment of women [3].

Violence against pregnant women seems to be more prevalent than diseases routinely investigated during prenatal care, such as pre-eclampsia and diabetes [4]. Literature reviews have shown rates ranging from 0.9% to 57.1% depending on the choice of methods and on sociocultural conditions [4-7]. In Brazil, the prevalence rates detected by researchers of the *Multi-Country Study* of the World Health Organization were 8% for the city of São Paulo and 11.1% for the Wooded Zone of Pernambuco [3]. Taillieu and Brownridge [4] stated that maltreatment of pregnant women was recurrent in successive pregnancies.

During the gestational period, rates and risk factors for psychological violence (more frequent than physical or sexual violence) have been less investigated [4,8], perhaps because the instrument more frequently used for screening, the Abuse Assessment Screen, only permits a detailed investigation of physical violence during pregnancy [4,9].

Prevalence rates of psychological violence during pregnancy ranged from 1.5% to 43.2%. The highest was detected in Pakistan [4]. Prevalence rate of psychological violence on the part of intimate partners on the African continent was 24.8% (Uganda), 41% (a public hospital in South Africa) and 49% (a rural community in South Africa) [7].

In Brazil, the highest rate (61.7%) was detected in puerperae admitted to the three major public maternities of Rio de Janeiro [10]. In the *Multi-country study*, psychological violence on the part of the intimate partner was the most frequent (28.8%), followed by physical (11.6%) and sexual violence (5.6%) [11].

Regarding the factors associated with violence during pregnancy, Tailleu and Brownridge [4] called attention to the fact that the analyses were almost always limited to the bivariate stage. A previous history of violence was pointed out by these researchers as one of the strongest predictors. Divorced/separated women were found to be at higher risk, even when confounding factors were controlled for, such as women who smoked, ingested alcohol and/or took illicit drugs [4]. Associations between violence during pregnancy and age range, race/ethnic group, educational level, remunerated job of the pregnant woman and of her intimate partners and family income yielded inconclusive results [4].

In Brazil, only one study investigated factors associated with domestic psychological violence during pregnancy. Pregnant women with up to eight years of schooling, economically responsible for the family, with common mental disorder and who had witnessed or suffered physical aggression before 15 years of age, who had intimate partners up to the age of 19 years and who ingested alcohol two or more times per week were at higher risk of suffering psychological violence [12]. The authors did not report whether they had included in their analysis pregnant women submitted also to other types of violence.

The present study was conducted in order to investigate the prevalence rate of psychological violence against pregnant women seen at prenatal care services in the municipality of São Luís, Brazil and to analyze factors associated with exclusive psychological violence (EPV) and recurrent exclusive psychological violence (REPV).

## Methods

From February 2010 to June 2011, 1446 pregnant women were interviewed in phase one (prenatal) of a cohort investigating new etiological factors for preterm birth in the municipality of São Luís (Maranhão/Brasil), which is part of the Brazilian Birth Cohort Studies of Ribeirão Preto and São Luís (Brisa).

Data was obtained in a convenience sample. It was impossible to draw a random sample of the population of pregnant women in São Luís because there was no list available. The inclusion criteria were: having performed the first ultrasound exam at less than 20 weeks of gestational age and to intend to give birth at one of the maternities in the municipality. Pregnant women carrying more than one fetus were not included.

Pregnant women attending prenatal care clinics of three public maternity hospitals and services of obstetric ultrasonography were invited to participate in an interview to be held at 22 to 25 weeks of gestational age.

In the analysis of factors associated with EPV, a minimum number of 948 women interviewed would be necessary. This sample size considered a 5% probability of type I error, 80% statistical power and prevalence of psychological violence against pregnant women of 11.1%^3^ (estimate based on the *Multi-country study* of the WHO, in the Wooded Zone of Pernambuco, Brazil).

To determine the prevalence rate of EPS, we excluded 199 pregnant women submitted also to physical and sexual abuse and four women who did not respond to one or more screening questions for these two types of violence. To determine the prevalence rates of REPV, i.e., abuse occurring on more than one occasion, we excluded women submitted to EPV on only one occasion. After the additional exclusion of 295 pregnant women who did not live with their intimate partners, the effective sample size was 982.

For the investigation of the factors associated with recurrent abuse, we excluded an additional 184 pregnant women who had been submitted to EPV only once, resulting in 1078 women.

Before the application of the questionnaire, the pregnant women gave written informed consent to participate in the study. When they were younger than 18 years, an accompanying adult also signed the consent form. It was explained to all women that the Brisa prenatal cohort was investigating possible causes of preterm birth, such as violence against pregnant women, and that confidentiality, image protection and non-stigmatization were guaranteed to all of them.

Data of interest were selected from a database containing information of the *Self-Applied Prenatal Questionnaire* and of the *Prenatal Interview Questionnaire*. Data about violence, social support and use of illicit drugs by the interviewees were obtained from the self-applied questionnaire. Some demographic, socioeconomic and behavioral characteristics of the pregnant women were obtained from the second questionnaire, in addition to life aspects of the intimate partners residing with the interviewees and of the family heads. The skin color variable was obtained from the *Birth Questionnaire*.

For the screening of psychological violence, we used the instrument of the *Multi-country study on women’s health and violence against women* of the WHO. The instrument *Social Support Scale* of the *Medical Outcomes Study* (MOS) was used to investigate the material dimension and the groups of affective/positive social interaction and emotional information. The two instruments have been validated for Brazil [13,14].

Psychological violence was considered to have occurred when the interviewee responded affirmatively to one of the four following questions: during this pregnancy, did anybody at any time a) Insult you or make you feel bad about yourself? b) Belittle of humiliate you in front of other persons? c) Do something to scare and intimidate you on purpose? d) Threaten to hurt you or somebody you like?

In the maternity hospitals of São Luís, 1379 puerperae of the cohort were interviewed a second time on the occasion of the birth of their children. Characteristics of the life of the women and their relatives and intimate partners were obtained, as well as data about childbirth and the neonate, born alive or stillborn. The data of the women who were not interviewed at the time of delivery were obtained at their home in case they were located. The chronological age of the pregnant women ranged from 14 to 45 years.

The theoretical-conceptual model was hierarchized at three levels. Level 1 included variables expressing demographic and socioeconomic characteristics and gender inequalities: a) age range of the pregnant woman and of her residing intimate partner (up to 19 years, 20 to 24 years or 25 years or more); b) self-reported skin color of the pregnant woman (white, black, crossbred – mixed/mulatto/cabocla/brown – or yellow/oriental); c) educational level of the pregnant woman and of her residing intimate partner (elementary, middle or higher); d) remunerated job of the pregnant woman (no or yes); e) family income in minimum wages (less than 1, 1 to less than 3, 3 to less than 5, or 5 or more); f) Brazilian economic classification (classes A/B, C or D/E, with classes A and B having the highest educational level and owning more consumer goods, class C being intermediate, and classes D and E having the lowest educational level and being the poorest) [15]; g) differences in schooling and occupation between the pregnant women and their residing intimate partners (no difference, pregnant women with higher schooling or better occupation, or partners with higher schooling or better occupation); and h) who is the head of the family, i.e., the person with the highest income (pregnant woman, intimate partner, or somebody else).

Level 2 analyses involved variables that demonstrate the social support received by the pregnant women: material, emotional/information and affective/positive social interaction dimensions, with cut-off points in the 75th percentile (inadequate when lower than the 75th percentile and adequate in the opposite case).

**Figure 1:**
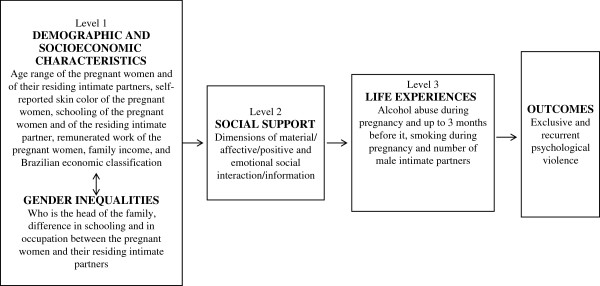
Hierarchical model for the assessment of factors associated with psychological violence.

Level 3 variables express life experiences of the pregnant women. The following variables were selected: a) alcohol abuse (four or more doses on a single occasion) during pregnancy (yes or no); use of illicit drugs during pregnancy and/or up to 3 months before it (yes or no); c) smoking during pregnancy (yes or no); and d) number of male partners with whom the pregnant women had sex relations during life (1, 2 to 5 or 6 or more intimate partners) (Figure 1).

Descriptive analysis was used to present frequencies and percentages. The Poisson regression model with robust adjustment of variance was used to investigate the associations between independent variables and outcomes because the prevalence rates of violence detected were higher than 10%.

The independent variables with a p-value of less than 0.2 in bivariate analysis were selected for adjusted analysis in each level of the hierarchical model. The category of the variable with the lowest percentage of violence was considered as reference. Prevalence ratio (PR) and its 95% confidence interval were used for the analysis of associated factors. Level 1 variables were first adjusted to each other and the variables with a P-value of less than 0.10 were selected for inclusion in the subsequent level. Level 2 variables plus the variables with a P < 0.10 value in the previous level were then adjusted simultaneously. Finally, level 3 variables plus variables with a P < 0.10 value in the previous levels were then adjusted simultaneously. The significance of each variable was considered at the level to which it belonged.

The investigation reported in the present paper fulfilled the requirements of Resolution 196/96 of the National Health Council and its complementary norms and was approved by the Research Ethics Committee of the University Hospital of the Federal University of Maranhão (protocol nº 4771/2008-30).

## Results

EPV prevalence rate was 41.6% and REPV prevalence rate was 32.6%.

With respect to EPV outcome, the results of the descriptive and bivariate analyses of gender inequalities, demographic and socioeconomic characteristics, social support received and life experiences are presented in Table 1.

**Table 1:** Bivariate analysis of the factors associated with exclusive psychological violence

	**Exclusive psychological violence**					
**Variables**	**n**	**%**	PR*	95 CI**		p***
Level 1						
Age range of the pregnant women (years) (n = 1.243)						0.076
25 or more	696	38.7	1			
20 to 24	393	45.0	1.16	1.01	1.34	
14 to 19	154	45.4	1.17	0.96	1.43	
Age range of the intimate partners (years, n = 1.001)						0.268
25 or more	734	37.6	1			
20 to 24	242	42.1	1.12	0.94	1.33	
14 to 19	25	48.0	1.28	0.84	1.94	
Skin color of the pregnant women (n = 1.181)						0.024
Crossbred	794	39.1	1			
Black	195	43.0	1.10	0.92	1.32	
White	171	46.7	1.19	0.99	1.43	
Yellow/Oriental	21	61.9	1.58	1.12	2.23	
Schooling of the pregnant women (n = 1.243)						
Elementary	148	39.1	1			0.157
Middle	944	40.8	1.04	0.84	1.29	
Higher	151	48.3	1.23	0.95	1.59	
Schooling of the residing intimate partner (n = 982)						0.499
Higher	79	36.7	1			
Middle	728	37.7	1.03	0.76	1.39	
Elementary	175	42.2	1.15	0.82	1.61	
Remunerated job of the pregnant women (n = 1.243)						0.331
No	643	40.2	1			
Yes	600	43.0	0.94	0.82	1.07	
Family income in minimum wages (n = 1.209)						0.094
<1	45	35.5	1			
1 to <3	687	39.3	1.10	0.74	1.66	
3 to <5	292	44.1	1.24	0.82	1.88	
≥5	185	48.1	1.35	0.89	2.06	
Brazilian economic class (n = 1.186)						0.549
C	811	40.4	1			
D/E	178	43.2	1.07	0.89	1.29	
A/B	197	44.1	1.09	0.91	1.30	
Who is the family head (n = 1.239)						0.072
Partner	746	39.0	1			
Pregnant woman	129	44.1	1.13	0.91	1.40	
Others	364	45.8	1.18	1.02	1.36	
Difference in schooling (n = 982)						0.050
Pregnant woman < residing intimate partner	92	30.4	1			
No difference	684	37.7	1.24	0.89	1.71	
Pregnant woman > residing intimate partner	206	44.6	1.47	1.04	2.07	
Difference in occupation (n = 992)						0.923
Pregnant woman < residing intimate partner	197	38.0	1			
No difference	158	38.6	1.01	0.78	1.32	
Pregnant woman > residing intimate partner	637	39.5	1.04	0.85	1.27	
Level 2						
Material dimension of social support <75th percentile (n = 1,243)						0.001
No	378	34.3	1			
Yes	865	44.7	1.30	1.11	1.52	
Positive affective/social interaction dimension of social support <75^th^ percentile (n = 1.243)						<0.001
No	351	31.9	1			
Yes	892	45.4	1.42	1.20	1.68	
Emotional dimension/information of social support <75th percentile (n = 1.243)						0.001
No	337	32.9	1			
Yes	906	44.8	1.36	1.14	1.61	
Level 3						
Smoking during pregnancy (n = 1,243)						0.009
No	1204	41.0	1			
Yes	39	58.9	1.43	1.09	1.88	
Use of illicit drugs up to 3 months before the current pregnancy (n = 1,240)						0.003
No	1224	41.3	1			
Yes	16	68.7	1.66	1.19	2.33	
Alcohol abuse by the woman during pregnancy (n = 1,241)						0.013
No	1129	40.5	1			
Yes	112	51.7	1.28	1.05	1.55	
Number of sex partners (n = 1.231)						0.001
1	370	36.7	1			
2 to 5	734	41.2	1.12	0.96	1.32	
6 or more	127	55.1	1.50	1.22	1.84	

*Prevalence Ratio.

**95% Confidence interval.

***P-value obtained by the likelihood ratio test based on the Poisson regression model.

São Luís-Brazil, 2010/2011.

In adjusted analysis, the following variables continued to be associated with EPV (Table 2): age of the pregnant woman of 14 to 19 years (PR: 1.32 95% CI: 1.04 - 1.70), higher educational level of the pregnant woman than that of her intimate partner (PR: 1.54, 95% CI: 1.09 - 2.16), inadequate affective social support/positive social interaction (PR: 1.34, 95% CI: 1.11 - 1.62), use of illicit drugs by the pregnant woman (PR: 1.80, 95% CI: 1.16 - 2.81), and having had six or more intimate partners in life (PR: 1.52, 95% CI: 1.18 - 1.96).

**Table 2:** Adjusted analysis of the factors associated with exclusive psychological violence

**Variables (n = 970)**	PR*	95 CI**		p***
Level 1				
Age range of the pregnant women (years)				0.037
25 or more	1			
20 to 24	1.18	0.99	1.40	
14 to 19	1.32	1.04	1.70	
Difference in schooling				0.020
Pregnant woman < residing intimate partner	1			
No difference	1.25	0.91	1.73	
Pregnant woman > residing intimate partner	1.54	1.09	2.16	
Level 2				
Positive affective/social interaction dimension of social support <75^th^ percentile				0.002
No	1			
Yes	1.34	1.11	1.62	
Level 3				
Smoking during pregnancy				0.066
No	1			
Yes	1.42	0.98	2.07	
Use of illicit drugs up to three months before the current pregnancy				0.009
No	1			
Yes	1.80	1.16	2.81	
Number of sex partners				0.003
1 partner	1			
2 to 5	1.09	0.90	1.31	
6 or more	1.52	1.18	1.96	

*Prevalence Ratio.

**95% Confidence Interval.

***P-value obtained by the likelihood ratio test based on the Poisson regression model.

São Luís-Brazil, 2010/2011.

The results of the descriptive and bivariate analyses of REPV outcome are summarized in Table 3.

**Table 3:** Bivariate analysis of the factors associated with recurrent exclusive psychological violence

	**Recurrent psychological violence**					
**Variables**	**n**	**%**	PR*	95 CI**		p***
Level 1						
Age range of the pregnant woman (years, n = 1.078)						0.136
25 or more	612	30.3	1			
14 to 19	125	32.8	1.08	0.82	1.42	
20 to 24	341	36.6	1.21	1.01	1.45	
Age range of the partner (years, n = 878)						0.324
25 or more	646	29.1	1			
20 to 24	211	33.6	1.16	0.92	1.48	
14 to 19	21	38.1	1.31	0.75	2.29	
Skin color of the pregnant woman (n = 1.022)						0.043
Crossbred	691	30.1	1			
Black	169	34.3	1.14	0.89	1.45	
White	145	37.2	1.24	0.97	1.57	
Yellow/Oriental	17	52.9	1.76	1.11	2.79	
Schooling of the pregnant woman (n = 1.078)						0.048
Elementary	810	31.1	1			
Middle	135	33.3	1.07	0.83	1.39	
Higher	133	41.3	1.33	1.06	1.67	
Schooling of the partner (n = 862)						0.493
Middle	637	28.8	1			
Higher	73	31.5	1.09	0.76	1.56	
Elementary	152	33.5	1.16	0.90	1.49	
Remunerated job of the pregnant woman (1.078)						0.714
No	566	32.1	1			
Yes	512	33.2	0.97	0.82	1.15	
Family income in minimum wages (n = 1,047)						0.380
<1	40	27.5	1			
1 to <3	604	30.9	1.12	0.67	1.89	
3 to <5	252	35.3	1.28	0.75	1.18	
≥5	151	36.4	1.32	0.77	2.29	
Brazilian economic class (n = 1.028)						0.071
C	692	30.2	1			
D/E	159	36.4	1.21	0.95	1.52	
A/B	177	37.8	1.25	1.01	1.56	
Who is the family head (n = 1.075)						0.152
Partner	655	30.5	1			
Pregnant woman	109	33.9	1.11	0.83	1.48	
Others	311	36.6	1.20	0.99	1.44	
Difference in schooling (n = 862)						0.137
Pregnant woman < residing intimate partner	83	22.8	1			
No difference	604	29.4	1.29	0.85	1.95	
Pregnant woman > residing intimate partner	175	34.8	1.52	0.97	2.37	
Difference in occupation (n = 868)						0.150
Pregnant woman < residing intimate partner	160	23.7	1			
No difference	565	31.8	1.34	0.99	1.82	
Pregnant woman > residing intimate partner	143	32.1	1.35	0.94	1.95	
Level 2						
Material dimension of social support <75th percentile (n = 1,078)						0.001
No	327	24.1	1			
Yes	751	36.3	1.50	1.21	1.86	
Affective dimension/positive social interaction of social support <75th percentile (n = 1,078)						0.001
No	311	23.1	1			
Yes	767	36.5	1.58	1.26	1.97	
Emotional dimension/social support information <75th percentile (n = 1,078)						0.001
No	294	23.1	1			
Yes	784	36.2	1.57	1.25	1.968	
Level 3						
Smoking during pregnancy (n = 1,078)						0.006
No	1045	32.0	1			
Yes	33	51.5	1.60	1.141	2.26	
Use of illicit drugs up to 3 months before the current pregnancy (n = 1.078)						0.018
No	1063	32.4	1			
Yes	12	58.3	1.79	1.10	2.92	
Alcohol abuse by the pregnant woman during pregnancy (n = 1,076)						0.008
No	980	31.5	1			
Yes	96	43.7	1.39	1.09	1.77	
Number of sex partners (n = 1,067)						0.002
1	330	29.0	1			
2 to 5	632	31.8	1.09	0.89	1.34	
6 or more	105	45.7	1.57	1.20	2.05	

* Prevalence Ratio.

** 95% Confidence Interval.

***P-value obtained by the likelihood ratio test based on the Poisson regression model.

São Luís-Brazil, 2010/2011.

In the adjusted analysis, factors associated with REPV were: inadequate social affective support/positive social interaction (PR: 1.47 95% CI: 1.15 – 1.87), use of illicit drugs by the pregnant woman (PR: 2.28 95% CI: 1.40 – 3.71) and having had six or more intimate partners during life (PR: 1.47 95% CI: 1.06 – 2.03) (Table 4).

**Table 4:** Adjusted analysis of the factors associated with recurrent exclusive psychological violence

**Variables (n = 868)**	PR*	95 CI**		p***
Level 2				
Affective dimension/positive social interaction of social support <75th percentile				0.002
No	1			
Yes	1.47	1.15	1.87	
Level 3				
Smoking during pregnancy				0.065
No	1			
Yes	1.54	0.97	2.44	
Use of illicit drugs up to 3 months before the current pregnancy				0.001
No	1			
Yes	2.28	1.40	3.71	
Number of sex partners				0.048
1	1			
2 to 5	1.04	0.83	1.30	
6 or more	1.47	1.06	2.03	

*Prevalence Ratio.

**95% Confidence Interval.

***P-value obtained by the likelihood ratio test based on the Poisson regression model.

São Luís-Brazil, 2010/2011.

## Discussion

EPV prevalence rate was 41.6% and REPV prevalence rate was 32.6%. Inadequate social affective support/positive social interaction, use of illicit drugs by the pregnant woman and having had six or more intimate partners during life were associated with both EPV and REPV. Being young or having higher educational level than their intimate partners were associated only with EPV.

Except for the results detected in the municipality of Rio de Janeiro (61.7%) [10], in a rural community in South Africa (49%) [16] and in the Southern Appalachians (79.8%) [17], the prevalence rate of psychological violence (48.4%) detected in the Brisa prenatal cohort was higher than those reported in other studies, which ranged from 1.5% to 43% [4,8,11,12,18-24]. EPV and REPV prevalence rates were lower than those reported in only four of these publications [10,16,17,19].

These disparities can be attributed to methodological and sociocultural differences, as demonstrated in literature reviews about violence against pregnant women [5-7]. It should be pointed out that the general objective of the Brisa prenatal cohort was to investigate new etiological factors for preterm birth, with the violence against pregnant women being one of the factors investigated. Since violence was not the main study question, this may have facilitated reports of violence by the women.

Three hypotheses can explain these higher rates in the present study: a) the use of a self-applied instrument without the presence of the interviewer; b) the interviewees were constantly reminded of the impossibility of being identified in their responses; and c) violence during pregnancy was being investigated as a risk factor for preterm birth, a fact that may have contributed to increased report of abuse suffered by them.

Factors associated with psychological violence against pregnant women have not been routinely analyzed, even when prevalence rates are estimated [8]. Conditions associated with psychological domestic violence or violence on the part of an intimate partner have been frequently analyzed without distinguishing them from other types of violence [4,6,7,16,19,22-25], especially from physical violence [10,18,20], a fact that impairs the comparability of the present result to those of previous studies.

In the Brisa prenatal cohort, EPV and REPV against pregnant women were not associated with skin color, educational level or working and economic variables. Also, they were not associated with characteristics of the intimate partners and heads of family, alcohol abuse or smoking.

EPV was observed more frequently among pregnant women aged 14 to 19 years. Although some studies have pointed out this age range as being of higher risk for violence, a review of the literature has revealed that it was not possible to make this statement since the samples were mostly collected at hospitals or clinics and were not population based [4]. The results of investigations conducted in Michigan and in the municipality of Campinas, Brazil have shown that there was no association between psychological abuse and age of less than 20 years [8,12]. Durand and Schraiber [24] also detected no association between psychological violence by intimate partners and age of the pregnant woman. However, these authors did not state whether they excluded pregnant women submitted to physical and/or sexual violence from their analyses.

Pregnant women with an educational level higher than that of their residing intimate partners may have more frequently challenged the norms of gender hierarchy than those with an educational level similar to, or lower than that of their husband/companion. The variable concerning difference in schooling between pregnant women and their residing intimate partners was elaborated for the present investigation and no equivalent variable was detected in publications investigating factors associated with violence against pregnant women [4,6-8,10,12,16-24].

A more usual analysis is that between violence by the intimate partner and women’s schooling. Literature reviews consider the results of studies investigating this association to be inconsistent [4] or controversial [7]. As was the case for the Brisa prenatal cohort, several investigations have not detected an association in adjusted analysis [19,23,24]. In addition, a cross-sectional study conducted on 3675 pregnant women in the state of Michigan (USA) did not detect an association between psychological abuse and having less than twelve years of study [8]. However, in the municipality of Campinas (São Paulo), Brazilian investigators interviewed 1379 pregnant women and observed a higher risk of psychological violence for pregnant women with up to eight years of study [12].

In the present study we observed that inadequate social affective support/positive social interaction was associated with EPV and REPV. More recently, inadequate social support has started to be investigated as a risk factor for violence against women. A review of the literature yielded results showing a lower occurrence of violence against pregnant women in the presence of an adequate network of social support [4]. The two studies that considered psychological violence as an outcome did not analyze the social support offered to pregnant women [8,12]. The affective dimensions (assessed with three questions)/positive social interaction (four questions) investigate the physical demonstrations of love and affection and being able to count with persons with whom to relax and have fun [14].

The association between violence and use of illicit drugs was also related to EPV and REPV and was detected in various studies analyzed in a review article [4]. The Brazilian study considering psychological violence as the outcome did not investigate the use of illicit drugs during pregnancy [12]. However, the study conducted in the North American state of Michigan revealed a higher risk for this type of violence for women who used illicit drugs (OR 2.17, 95% CI 1.39; 3.37) [8]. Charles and Perreira [18] did not detect an association between this variable and psychological/physical violence by an intimate partner. Stress preceding pregnancy may have been a justification for the use of illicit drugs [4].

Having had six or more intimate male partners in life was associated with the two outcomes. From this perspective, a systematic review revealed a higher risk of violence for pregnant women with more than five intimate partners during their lives [7]. The only two studies identified that considered psychological violence as the outcome, did not investigate the variable number of intimate partners [8,12]. Being aware that his wife/companion/girl friend had other male partners may represent for an intimate partner his lack of control on the body of the woman, resulting in violence [4].

Finally, limitations of the present study should be pointed out, summarized as follows: a) the study was cross-sectional, a design that does not permit to establish cause-effect relationships; b) data about non-residing intimate partners were not collected; and c) this was a convenience sample. As strong points of the study we may mention that this was a population study with a large sample size, which innovated when pregnant women submitted to physical and sexual violence were excluded from the analysis of factors associated with psychological violence. Difficulties in reading and writing have been identified as one of the limitations of the self-administered questionnaire. Furthermore, violence rates tend to be lower when using a self-administered questionnaire [4].

## Conclusions

The rates of psychological violence were higher than those reported in other studies conducted in Brazil and in other countries. There was a greater risk of psychological violence for adolescent women and also for those who challenged hierarchical gender norms since this violence was associated with higher educational level of the interviewee compared to her residing intimate partner and with her having had six or more male intimate partners in life. Pregnant women with an inadequate affective social support/positive social interaction were submitted more frequently to psychological violence and to more numerous episodes of this type of abuse. Unhealthy practices such as the use of illicit drugs during pregnancy and/or up to three months before was a risk factor for psychological violence. There is a need for routine investigation of psychological abuse of pregnant women during their prenatal visits at health care services. Women should also be instructed to demand services of assistance to women victims of psychological violence.

## Competing interests

The authors declare that there are no conflicts of interest regarding political, economic, ideological, religious, academic, intellectual, and personal or any other aspects.

## Authors’ contributions

MR, AS, MA, RB, MB and HB were involved in the design and implementation of the study including data collection. All authors have been involved in the analyses, writing, or combinations of these activities and have approved the final manuscript.

## Authors’ information

AS^2^ and MB^7^ coordinated the Brazilian Birth Cohort Studies of Ribeirão Preto and São Luís (Brisa). RB^2^ and HB^7^ coordinated the field activities. MA^2^ supervised the data collection. LR^3^ investigates gender violence. LS^4^ coordinated the *Multi-country study* of the World Health Organization in Brazil.

## Pre-publication history

The pre-publication history for this paper can be accessed here:

http://www.biomedcentral.com/1471-2393/14/66/prepub
